# Changes in emotions and worries during the Covid-19 pandemic: an online-survey with children and adults with and without mental health conditions

**DOI:** 10.1186/s13034-021-00363-9

**Published:** 2021-02-21

**Authors:** Josefine Rothe, Judith Buse, Anne Uhlmann, Annet Bluschke, Veit Roessner

**Affiliations:** Department of Child and Adolescent Psychiatry, Faculty of Medicine, Technische Universität Dresden, Fetscherstrasse 74, 01307 Dresden, Germany

**Keywords:** Psychosocial stress, Crisis, SARS-CoV-2, Family, Lockdown

## Abstract

**Background:**

The novel coronavirus disease (Covid-19) has spread quickly worldwide with dramatic consequences on our daily lives. Adverse psychosocial consequences of Covid-19 might be particularly severe for children and adolescents, parents of young children and people with mental health conditions (mhc), who are more prone to the experience of psychosocial stress and who are more dependent on the access to professional psychosocial support. The present survey therefore aimed to explore perceived stress and the emotional responses of children and adolescents as well as adults with and without mhc during the social restrictions due to the Covid-19 pandemic.

**Methods:**

The survey gathered information about 284 children and adolescent (parent-on-child-reports) and 456 adults (including 284 parents, self-reports). The participants were allocated to four groups: children and adolescents with mhc, children and adolescent without mhc, adults with mhc and adults without mhc. The survey included general questions about socio-demographic characteristics and mental health status, the CoRonavIruSHealth Impact Survey and the Perceived Stress Scale (only data on adults). Wilcoxon signed-rank tests were used for comparing the emotional responses during the Covid-19 pandemic with emotions before the Covid-19 pandemic. Independent sample t-test were used to compare the level of perceived stress between the adult groups, linear regression analyses were conducted to examine which variables predicted perceived stress during the Covid-19 restrictions.

**Results:**

An increase to the worse during the Covid-19 restrictions was observed for most emotions and worries in all four groups (children and adolescents with mhc, children and adolescents without mhc, adults with mhc, adults without mhc). Contrary to our expectations, a greater number of emotions worsened significantly for children and adolescents as well as adults without mhc as compared to those with mhc. We found higher perceived stress in parents as compared to adults without children in the same household and in adults with mhc as compared to those without mhc.

**Discussion:**

Covid-19-related social restrictions and potential health risks seem to affect emotions and perceived stress in children, adolescents and adults. Especially, Covid-19 seems to be have worsened the mental well-being of children and adolescent and their families, who were mentally healthy before the Covid-19 pandemic.

## Introduction

The novel coronavirus disease (Covid-19) has spread quickly worldwide with dramatic consequences on our daily lives. All over the world, public life has been severely restricted to slow down the spread of the virus. Universities, schools and kindergartens were closed, leaving home was strongly restricted, meeting friends and relatives was prohibited as was travel at the national and international level. It is well known that a restriction to free movement connected to social isolation is one of the main features that influences psychological well-being. From previous serious but not as widespread epidemics (e.g. Ebola, SARS, HIV) we know of adverse psychosocial consequences not only for the infected but also for the non-infected population [1, 2]. For Covid-19, there are initial findings on the psychosocial consequences for the general population [3–7]. For example, in an online survey (N_total_ = 1639) 48.2% of the respondents showed a low psychological well-being (raw scores ≤ 60 on the Psychological General Well-Being questionnaire with a maximum raw score of 110; [8]) linked to Covid-19 spreading [4].

While adverse psychosocial consequences of Covid-19 can be observed anywhere in the general population, the negative effects might be particularly severe for people, who are more prone to the experience of psychosocial stress, and for those, who are more dependent on the access to professional psychosocial support, as this is the case in people with mental health conditions (mhc) and in children and adolescents and their parents.

For families with children or adolescents with mental health needs it is additionally challenging that access to mental health support is cancelled or limited, and remote online or telephone support cannot fully substitute face-to-face contacts. Asbury and colleagues [9] asked parents of children with special educational needs and disabilities in the UK to describe (free response question) how the Covid-19 outbreak affected their own mental health and the mental health of their child. Many parents reported loss of children's routine (35%), loss of friends and children's community (12%), and loss of specialist input (11%) as a burden on all family members. Pisano, Galimi and Cerniglia [10] found high rates of increased irritability (53.53%) and increased laziness (43.26%) in 4–10 years old Italian children during Covid-19. Examining the “interplay between mothers’ and children’s behavioral and psychological factors during COVID-19” in an Italian sample, Di Giorgio and colleagues [11] noted that mothers reported an increase in emotion symptoms, conduct problems and hyperactivity/inattention issues in their children, assessed with the Strengths and Difficulties Questionnaire (parent version). Interestingly, this finding was independent of the mothers’ working status during COVID-19 (regular working, not working, stopped working, working at home). In contrast, the individual and dyadic stress of the parents mediated the quarantine’s impact (difficulties experienced by parents during the quarantine) on behavioral and emotional problems of the child significantly [12]. Further evidence for the mediating role of perceived stress comes from Ceram and colleagues [3], who reported that among adult Italian residents, about 48% of the total effect of loneliness on the perceived health impact of Covid-19 was mediated by perceived stress.

During Covid-19, most children and adolescents were home-schooled by their parents with no or only limited support from trained teachers. In addition to home-schooling their children, most parents needed to meet their occupational duties at the same level as before Covid-19, a situation that most likely provokes mental overload and in turn high levels of psychosocial stress. Parental stress in turn covaries with child behavior problems [13] and even impacts obesity in children [14].

The present survey aimed to explore emotional responses of children, adolescents and adults with and without mhc exposed to the social restrictions of the Covid-19 pandemic as well as perceived stress in the parents and in adults without children. We hypothesized that all survey participants would experience an increase to the worse regarding emotions and worries during the Covid-19 pandemic as compared to the time before Covid-19. We further hypothesized that the increase to the worse of emotions and worries would be greater in children, adolescents and adults with mhc as compared to those without mhc.

With regard to perceived stress we assumend higher levels in parents (as compared to adults without children) and in children, adolescents and adults with mhc. Taking an exploratory approach, we ran regression analyses assessing the contribution of prior individual characteristics (e.g. age, number of minors in the same household, mental and physical state before the pandemic) and psychosocial changes due to the Covid-19 pandemic (e.g. financial worries related to Covid-19 outbreak and changes in the quality of relationships with family and friends) to the perceived stress of adults during the Covid-19 restrictions.

## Methods

### Situation in Germany during data collection

The online survey was administered from April 4th to May 6th 2020. At that time, all schools and kindergartens in Germany had been closed for 3 weeks and physical distancing (minimum distance of at least 1.5 m; moving in public space unaccompanied or with only one other person) had been compulsory for 2 weeks. In addition, harder restrictions were applied in two federal states of Germany, Saxony and Bavaria (leaving home only for occupational, shopping and medical purposes). These federal restrictions concerned over 90% of the respondents. The SARS-CoV-2 infections increased exponentially in the 2 weeks before the survey (70,000 new cases within the last 2 weeks in Germany). At the start of the survey (April 4th), the number of cases in Germany exceeded the 100,000 mark but hospitals still had capacity and the death rates increased comparatively slowly. However, media coverage was dominated by the dramatic circumstances in other countries and Germany seemed to be in a waiting position of a threatening situation.

### Participants

Families of patients of the Clinic for Child and Adolescent Psychiatry and Psychotherapy of the University Hospital Dresden, families of previous study participants as well as young adult participants of previous studies were invited to take part in the survey by email. Our department conducts studies on various disorders in the field of child an adolescence psychiatry (e.g. ADHD, autism, chronic tic disorder and Tourette, eating disorders) both with affected as well as with healthy individuals. Therefore, a broad spectrum of individuals was invited to participate in the survey. Parents were invited to respond in the survey concerning both their child and themselves. Initially, parents were invited to answer the survey for their child who was a patient in our department and/or had participated in a previous study. If parents wanted to respond for more than one child, they could start separate surveys for each child. However, only one of the participating parents did this (thus, no correction of analyses was required). Hence, responses were collected for four groups: children and adolescents with and without mhc as well as adults with and without mhc. After participants had provided informed consent, they answered some general questions concerning socio-demographic characteristics and mental health status. Thereafter, they completed a questionnaire corresponding to the CoRonavIruSHealth Impact Survey v.01 (CRISIS) of the National Institutes of Health and the 10 item version of the Perceived Stress Scale (PSS-10; [15]). The survey was conducted according to the recommendations of the Helsinki Declaration. A total of 284 children and adolescents and 456 adults took part in the survey. Among the 456 adults were 284 parents who answered the questionnaires for both their child and themselves. Sample characteristics are displayed in Table 1.

**Table 1:** Sample characteristics

	Adult (self-report) (N = 456)		Child or adolescent (parent-report)	
	With mental health condition (N = 70)	Without mental health condition (N = 386)	With mental health condition (N = 111)	Without mental health condition (N = 173)
Sex, frequency (male/female/other)	4/66/0	77/309/0	57/53/1	98/75/0
Mean age (SD)	39.11 (8.34)	39.09 (10.22)	11.74 (3.99)	9.93 (3.76)
Age range	19–58	18–69	2–17	1–17
Perceived stress scale, mean t-value (SD)	65.50 (15.03)	53.77 (11.93)	–	–
Frequencies of mental disorders				
ADD/ADHD	–	–	25	–
Anxiety disorders	6	–	10	–
Autism	1	–	11	–
Borderline	3	–	–	–
Conduct disorders	–	–	5	–
Disruptive Mood Dysregulation Disorder	–	–	2	–
Eating disorders	2	–	15	–
Intellectual disabilities	–	–	1	–
Mood disorders	53	–	17	–
Nocturnal enuresis	–	–	1	–
OCD	2	–	8	–
Reaction to severe stress/adjustment disorders	7	–	14	–
Reactive attachment disorder	–	–	2	–
Selective mutism	–	–	2	–
Schizophrenia	1	–	–	–
Sleep disorder	–	–	1	–
Tic-Disorders	–	–	7	–
Trichotillomania	–	–	1	–
Unspecified disorder of psychological development	–	–	1	–
Not reported	4	–	7	–

### Measures

#### CoRonavIruSHealth Impact Survey (CRISIS)

The CRISIS questionnaires were developed “through a collaborative effort between the research teams of Kathleen Merikangas and Argyris Stringaris at the National Institute of Mental Health”.

The questionnaires record health and exposure status to Covid-19 as well as life changes due to Covid-19 in the previous 2 weeks. Furthermore, questions about daily behaviours, emotions and worries as well as media and substance use were surveyed using a 5-point likert scale concerning both the current status (during the last 2 weeks, i.e. during the Covid-19 pandemic) and the pre-Covid-19 status (during the last 3 months). For the present analysis, questions on emotions and worries of the last 3 months and 2 weeks were used (How worried were you/your child generally?, How happy versus sad were you/your child?, How relaxed versus anxious were you/your child?, How fidgety or restless were you/your child?, How fatigued or tired were you/your child?, How well were you/your child able to concentrate or focus?, How irritable or easily angered were you/your child?, How lonely were you/your child?, To what extent have you/your child had negative feelings?). In addition, reports on the general mental and physical state before the pandemic, financial worries related to Covid-19 outbreak, and changes in the quality of relationships with family and friends were assessed.

### Perceived Stress Scale (PSS-10)

To assess current stress levels, the well-established Perceived Stress Scale [15] was used. The scale measures whether life situations are classified as stressful on a 5-point response scale. For the German version of the 10-item scale (maximum score = 40) good internal consistency (Cronbach alpha = 0.84) was reported [16]. For analysis, t-scores were used, based on norms of the Harris Poll that gathered information using the PSS-10 with 2387 respondents in the United States [17]. The PSS-10 was only collected as self-report of adults (see Table 1).

### Statistical analysis

Priori power analyses were conducted using G*Power 3.1 [18]. Depending on the test applied (t-test, Wilcoxon signed-rank test, Mann–Whitney-U-test) the required total sample size ranged from 120 to 228 with a medium effect size (d = 0.50) and a power of 0.80.

The data on the ten emotions and worries were measured by a 5-point likert scale (so they are ordinal scaled) and do not show a normal distribution in the four investigated groups (children and adolescents with mhc, children and adolescents without mhc, adults with mhc, adults without mhc). Therefore Wilcoxon signed-rank tests and Mann–Whitney-U-test were used for comparisons of those emotion and worries before (last 3 month) and during the Covid-19 outbreak (last 2 weeks) in all four groups. As ten comparisons were run for each group, we use Bonferroni adjusted alpha levels (0.05/10 = 0.005).

An independent sample t-test was conducted to investigate the difference in the t-scores of perceived stress measure (PSS-10) between adults with and without mhc. A further independent sample t-test was conducted to investigate the difference in perceived stress between adults where at least one minor (younger than 18 years) lives in the same household (also referred to as parents) and adults without a minor in the same household. Considering that t-tests were robust to violations of normality, we examined the difference in perceived stress using t-tests, even though the data of the four examined groups show significant results on the Shapiro–Wilk test (adults with mhc: *W* = 0.96, *p* = 0.02; adults without mhc: *W* = 0.97, *p* < 0.01; parents: *W* = 0.97, *p* < 0.01; adults without minor in household: *W* = 0.96, *p* < 0.01). Linear regression analyses were conducted to examine whether age, number of minors in the same household, mental and physical state before the pandemic (measured by self-report), financial worries related to Covid-19 outbreak and changes in the quality of relationships with family and friends (measured by self-report) predict the perceived stress (t-scores) of adults during Covid-19 restrictions. Separate regression analyses were run for adults with and without mhc.

## Results

### Differences in emotions and worries before and during Covid-19 restrictions

During Covid-19, an increase to the worse was observed for most emotions and worries in all four groups (children and adolescents with mhc, children and adolescents without mhc, adults with mhc, adults without mhc). Data on response frequencies are shown in Additional files 1, 2. Among children and adolescents without mhc, Wilcoxon signed-rank tests indicated a significant increase in five out of the ten emotions and worries (worried, happy versus sad, enjoy activities, fatigued or tired, lonely). For children and adolescents with mhc three out of ten emotions and worries (enjoy activities, fatigued or tired, lonely) worsened significantly. In adults without mhc, a series of Wilcoxon signed-rank tests indicated that this increase was significant in eight out of ten captured emotions and worries (all except fatigued or tired and negative thoughts). In adults with mhc, the increase was significant for two out of the ten captured emotions and worries (worried, enjoy activities). Means and Standard deviations of emotions and worries are shown in Table 2. Results of the Wilcoxon signed-rank tests are shown in Table 3.

**Table 2:** Means and Standard deviations of emotions and worries before and during Covid-19 separated for the four groups (adults and children with and without mhc)

	Adults with mhc (N = 70)		Adults without mhc (N = 386)		Children with mhc (N = 111)		Children without mhc (N = 173)	
	M_before_ (SD)	M_during_ (SD)	M_before_ (SD)	M_during_ (SD)	M_before_ (SD)	M_during_ (SD)	M_before_ (SD)	M_during_ (SD)
Worried	2.14 (1.11)	3.06 (1.09)	1.74 (0.83)	2.58 (0.96)	2.01 (1.08)	2.12 (1.03)	1.41 (0.71)	1.99 (0.84)
Enjoy activities	2.76 (1.04)	3.40 (1.27)	2.04 (0.88)	2.92 (1.09)	2.57 (1.11)	3.13 (1.09)	1.75 (0.77)	2.80 (1.14)
Concentrated	2.83 (1.17)	3.20 (1.20)	2.16 (0.98)	2.49 (1.04)	3.28 (1.29)	3.21 (1.24)	2.71 (1.27)	2.66 (1.18)
Lonely	1.96 (1.07)	2.27 (1.27)	1.42 (0.76)	1.73 (1.01)	1.77 (0.94)	2.44 (1.13)	1.38 (0.62)	2.02 (0.98)
Negative thoughts	3.26 (0.99)	3.20 (1.10)	2.44 (0.92)	2.54 (0.99)	2.62 (1.29)	2.48 (.20)	1.87 (0.84)	1.93 (0.86)
Happy or sad	3.34 (0.92)	3.44 (1.09)	2.30 (0.94)	2.72 (0.94)	2.97 (1.08)	2.98 (1.03)	2.05 (0.90)	2.42 (0.90)
Relaxed or anxious	3.21 (1.05)	3.33 (1.10)	2.37 (0.97)	2.69 (1.03)	3.11 (1.03)	3.02 (0.97)	2.27 (1.03)	2.30 (0.97)
Fidgety or restless	2.29 (1.17)	2.39 (1.21)	1.67 (0.86)	1.85 (0.94)	2.59 (1.22)	2.58 (1.28)	2.09 (1.07)	2.11 (1.14)
Fatigued or tired	3.27 (1.05)	3.30 (1.15)	2.38 (0.89)	2.38 (1.03)	2.28 (1.19)	1.90 (1.02)	1.90 (0.87)	1.68 (0.87)
Irritable or easy angered	2.54 (0.97)	2.84 (1.14)	1.97 (0.86)	2.19 (1.01)	2.83 (1.11)	2.74 (1.11)	2.10 (1.02)	2.26 (1.06)

**Table 3:** Wilcoxon test comparing emotions and worries before and during Covid-19 separated for the four groups (adults and children with and without mhc)

	Adults with mhc (N = 70)			Adults without mhc (N = 386)			Children with mhc (N = 111)			Children without mhc (N = 173)		
	*Z*	*p*	*r*	*Z*	*p*	*r*	*Z*	*p*	*r*	*Z*	*p*	*r*
Worried	− 4.83	≤ .005	.58	− 11.95	≤ .005	.61	− 1.11	.27	.11	− 6.41	≤ .005	.49
Enjoy activities	− 3.26	≤ .005	.39	− 11.23	≤ .005	.57	− 4.26	≤ .005	.40	− 8.29	≤ .005	.63
Concentrated	− 2.54	.01	.30	− 5.53	≤ .005	.28	− .61	.54	.06	− .52	.60	.04
Lonely	− 2.00	.05	.24	− 6.66	≤ .005	.34	− 5.28	≤ .005	.50	− 6.77	≤ .005	.51
Negative thoughts	− .28	.78	.03	− 2.16	.03	.11	− 1.93	.05	.18	− .89	.37	.07
Happy or sad	− .66	.51	.08	− 7.32	≤ .005	.37	− 1.89	.85	.02	− 4.56	≤ .005	.35
Relaxed or anxious	− .44	.66	.05	− 5.01	≤ .005	.25	− .87	.39	.08	− .41	.69	.03
Fidgety or restless	− .74	.46	.09	− 3.87	≤ .005	.20	− .41	.68	.04	− .17	.87	.01
Fatigued or tired	− .28	.78	.03	− .05	.96	.00	− 3.67	≤ .005	.35	− 2.96	≤ .005	.22
Irritable or easy angered	− 2.27	.02	.27	− 4.34	≤ .005	.22	− .92	.36	.09	− 2.15	.03	.16

Bonferroni adjusted alpha levels (0.05/10 = 0.005) were used

### Differences in emotions and worries between individuals with and without mhc

With regard to emotions and worries in children and adolescents, Mann–Whitney-U-tests indicated that children with mhc had worse values as compared to children without mhc in all captured emotions and worries except for fatigued or tired at the time before the Covid-19 restrictions. During the Covid-19 restrictions parental reports indicated worse values in children with mhc as compared to children without mhc in all captured emotions and worries except for worried, enjoy activities and fatigued or tired. A series of Mann–Whitney-U-tests indicated that adults with mhc had increased ratings in all ten captured emotions and worries both before and during the Covid-19 restrictions as compared to adults without mental health. Means and Standard deviations of emotions and worries are shown in Table 2. Results of the Mann–Whitney-U-tests are shown in Table 4.

**Table 4:** Mann–Whitney-U-test comparing emotions and worries of adults with and without mhc as well as children with and without mhc separated in before and during Covid-19

	Adults before Covid-19			Adults during Covid-19			Children before Covid-19			Children during Covid-19		
	*Z*	*p*	*r*	*Z*	*p*	*r*	*Z*	*p*	*r*	*Z*	*p*	*r*
Worried	− 2.78	≤ .005	0.13	− 3.48	≤ .005	0.16	− 5.06	≤ .005	0.30	− 0.77	.443	0.05
Enjoy activities	− 5.54	≤ .005	0.26	− 3.28	≤ .005	0.15	− 6.53	≤ .005	0.39	− 2.35	.019	0.14
Concentrated	− 4.39	≤ .005	0.21	− 4.68	≤ .005	0.22	− 3.54	≤ .005	0.21	− 3.64	≤ .005	0.22
Lonely	− 4.51	≤ .005	0.21	− 3.56	≤ .005	0.17	− 3.80	≤ .005	0.23	− 3.09	≤ .005	0.18
Negative thoughts	− 6.11	≤ .005	0.29	− 4.70	≤ .005	0.22	− 4.84	≤ .005	0.29	− 3.67	≤ .005	0.22
Happy or sad	− 7.82	≤ .005	0.37	− 5.37	≤ .005	0.25	− 6.87	≤ .005	0.41	− 4.40	≤ .005	0.26
Relaxed or anxious	− 5.98	≤ .005	0.28	− 4.48	≤ .005	0.21	− 6.10	≤ .005	0.36	− 5.58	≤ .005	0.33
Fidgety or restless	− 4.31	≤ .005	0.20	− 3.56	≤ .005	0.17	− 3.43	≤ .005	0.20	− 3.04	≤ .005	0.18
Fatigued or tired	− 6.49	≤ .005	0.30	− 6.15	≤ .005	0.29	− 2.35	.019	0.14	− 1.72	.085	0.10
Irritable or easy angered	− 4.57	≤ .005	0.21	− 4.55	≤ .005	0.21	− 5.28	≤ .005	0.31	− 3.63	≤ .005	0.22

Bonferroni adjusted alpha levels (0.05/10 = 0.005) were used

### Differences in perceived stress

The t-test for independent samples revealed that higher perceived stress levels during Covid-19 were found in parents (N = 324, M = 56.66, SD = 13.39) compared to adults not living with a minor (N = 132, M = 52.92, SD = 12.14, t (454) = − 2.78, p < 0.01). Furthermore, adults with mhc showed a significantly higher perceived stress level during Covid-19 than adults without mhc (t (85.474) = 6.18, p < 0.001).

### Predictors of perceived stress

Using the enter method, we found that the model explains a significant amount of the variance of perceived stress in adults with mhc (F(7,62) = 5.33, p < 0.001, R^2^ = 0.38, R^2^_Adjusted_ = 0.31). However, only physical condition reached significance as a predictor of perceived stress. For adults without mhc, the regression model also explained a significant amount of the variance of perceived stress (F(7,378) = 13.56, p < 0.001, R2 = 0.20, R2Adjusted = 0.19). Mental condition, relationship with family, relationship with friends, financial worries and number of minors in the same household were identified as significant predictors of perceived stress. Results of the regression coefficients are shown in Table 5.

**Table 5:** Regression of predictors of perceived stress (T-Value) for adults with and without mhc

*Predictor*	With mhc (N = 70)						Without mhc (N = 386)					
	*B*	*SE B*	*β*	*t*	*p*	*Adjusted R*^*2*^	*B*	*SE B*	*β*	*t*	*p*	*Adjusted R*^*2*^
1						.31						.19
Age	− .39	.21	− .22	− 1.89	.07		− .04	.06	− .03	− .68	.50	
Mental condition	4.03	2.21	.23	1.82	.07		3.02	.81	.20	3.73	< .01	
Physical condition	4.74	2.07	.29	2.29	< .05		1.05	.82	.07	1.28	.20	
Minors in same household	.70	1.29	.06	.54	.59		1.30	.52	.12	2.53	< .05	
Relationship family	.10	1.85	.01	.06	.96		3.17	.72	.21	4.43	< .01	
Relationship friends	5.48	2.84	.21	1.93	.06		3.81	1.04	.17	3.65	< .01	
Financial worries	1.42	1.40	.12	1.02	.31		1.93	.61	.15	3.15	< .01	

## Discussion

The present project provides insights into the different emotional responses of children, adolescents and adults (parents and non-parents) with and without mhc to the Covid-19-related social restrictions and potential health risks. As hypothesize, all captured emotions and worries (in adults) or most of the captured emotions and worries (in children) were worse in people with mhc as compared to people without mhc, both before and during the Covid-19 restrictions. In line with that, we found higher perceived stress in parents as compared to adults without children and in people with mhc as compared to people without mhc. Also as expected, our results indicate significant changes in emotions and worries from the time before the Covid-19 restrictions to the time during the Covid-19 restrictions in all four groups (children and adolescents with or without mhc, adults with or without mhc). The strongest increase among children was seen in ˈHow much did your child enjoy activitiesˈ and ˈHow lonely was your child?’. The strongest increase among both groups of adults was seen in ˈHow worried were you?’ and ‘How much did you enjoy activities?’. Interestingly and contrary to our expectations, a greater number of emotions worsened significantly for people (children and adolescents as well as adults) without mhc as compared to those with mhc. In correspondence with that, changes in the quality of relationships with family and friends as well as financial worries predicted perceived stress only in adults without mhc. In adults with mhc, physical condition before Covid-19 was the only predictor of perceived stress.

Our findings of worsened emotions and worries during Covid-19 restrictions in children, adolescents and adults without mhc are consistent with previous studies reporting a link between larger social networks and a better psychological well-being [19–21]. Our findings also match results from research on post-traumatic stress disorder (PTSD). For example, a meta-analysis has shown that lack of social support after trauma (e.g. from family and friends) and prior psychological adjustment were predictors of PTSD [22]. Furthermore, financial loss associated with a natural disaster has also been identified as a predictor of PTSD [23].

However, our findings raise the question why a smaller number of emotions worsened in children, adolescents and adults with mhc and why changes in the quality of relationships with friends and family were not predictors of perceived stress in the group of adults with mhc.

One explanation might be that individuals with mhc often have smaller friends and family networks or have strained family relationships (e.g. [24]). Therefore those individuals might have experienced less negative changes due to the Covid-19-related social restrictions. It also needs to be considered that the percentage of females was higher in the group of participants with mhc (94.28% as compared to 80.05%) and that the impact of family networks differs between females and males. In males, family networks are associated with a better well-being, while in females family networks can also place more obligations and burdens on them [19]. Another explanation for our findings might be that individuals with mhc have learned to develop strategies in order to cope with strained family relationships, e.g. with professional support. These strategies can include daily routines, sports or music. Stressful family situations and how to deal with them might therefore already be known to some people with mhc. This could be one reason why the change in family relationships as well as the number of minors in the same household have not predicted the perceived stress for people with mhc. Individuals without mhc usually cannot fall back on such a conscious dealing with stressful family situations, so changes in family relationships predicted the perceived stress significantly.

Another possible explanation for our finding that more emotions worsened significantly for individuals (children and adolescents as well as adults) without mhc as compared to those with mhc might be that social contacts are often fear-laden in individuals with mhc. The avoidance of social contacts in order to avoid negative social appraisal is a known characteristic of e.g. social anxiety disorder and depression. Moreover, performance anxiety is often observed in children and adolescents with mhc. Adolescents in a clinical sample reported higher levels of academic stress than adolescents in a non-clinical sample [25]. In those individuals, the avoidance of attending school might reduce their individual stress levels—a strategy that is usually not applicable in Germany with compulsory school attendance. While avoidance strategies are effectively maladaptive in long-term, because they entail a maintenance of the disorder, they often lead to a short-term relief from fears and anxiety. From this point of view, for individuals with mhc the Covid-19 related social restrictions and school closing might have served the purpose of an avoidance strategy with the short-term relief from social and performance anxiety. It is conceivably this short-term relief that has contributed to the finding that children, adolescents and adults did not show the expected extent of worsening of emotions and worries during Covid-19. However, since avoidance strategies are usually maladaptive in terms of maintaining the disorder in long-term, it would be very interesting to observe how emotions and worries of children and adolescents with mhc develop while Covid-19 restrictions endure.

## Conclusion

In sum, Covid-19-related social restrictions and potential health risks seem to affect emotions and worries of a large part of the population. Therefore, it will be important to observe trajectories of mhc in the general population. The trajectories could provide indications of factors that favour emotional recovery, and which may lead to chronic stress in such a physical distancing situation as the Covid-19 pandemic.

## Limitations and strengths of the study

A main limitation of the present study is that emotions and worries before Covid-19 were measured retrospective and therefore the recall bias might affected our results. The data is also limited in that the emotions and worries of children and adolescents are measured by parental reports. Furthermore, due to the small groups of mental disorders, the data are not suitable to provide information on how emotions and worries change in different mental disorders. Also, there was a special situation in Germany during data collection, whereby only a limited comparison with other studies is possible. It is moreover important to consider that the situation caused by Covid-19 has very specific characteristics and can therefore not easily be transferred to other shared experiences such as natural disasters or to individual tragedies such as accidents.

## Supplementary Information

**Additional file 1.** Frequencies of emotions and worries in adults. Frequencies of responses to all questions on emotions and worries in adults with and without mental health conditions (self-report).

**Additional file 2.** Frequencies of emotions and worries in children. Description of data: Frequencies of responses to all questions on emotions and worries in children with and without mental health conditions (parent-on-child-report).

## Data Availability

The datasets used and/or analysed during the current study are available from the corresponding author on reasonable request.

## Abbreviations

Covid-19: Coronavirus disease
CRISIS: CoRonavIruSHealth Impact Survey
PSS-10: Perceived Stress Scale
