# Supplementary material for: Changes in emotions and worries during the Covid-19 pandemic: an online-survey with children and adults with and without mental health conditions
Source: Child Adolesc Psychiatry Ment Health. 2021 Feb 21;15:11. doi: 10.1186/s13034-021-00363-9 (PMC7897360; doi:10.1186/s13034-021-00363-9)

a) How worried were you generally?

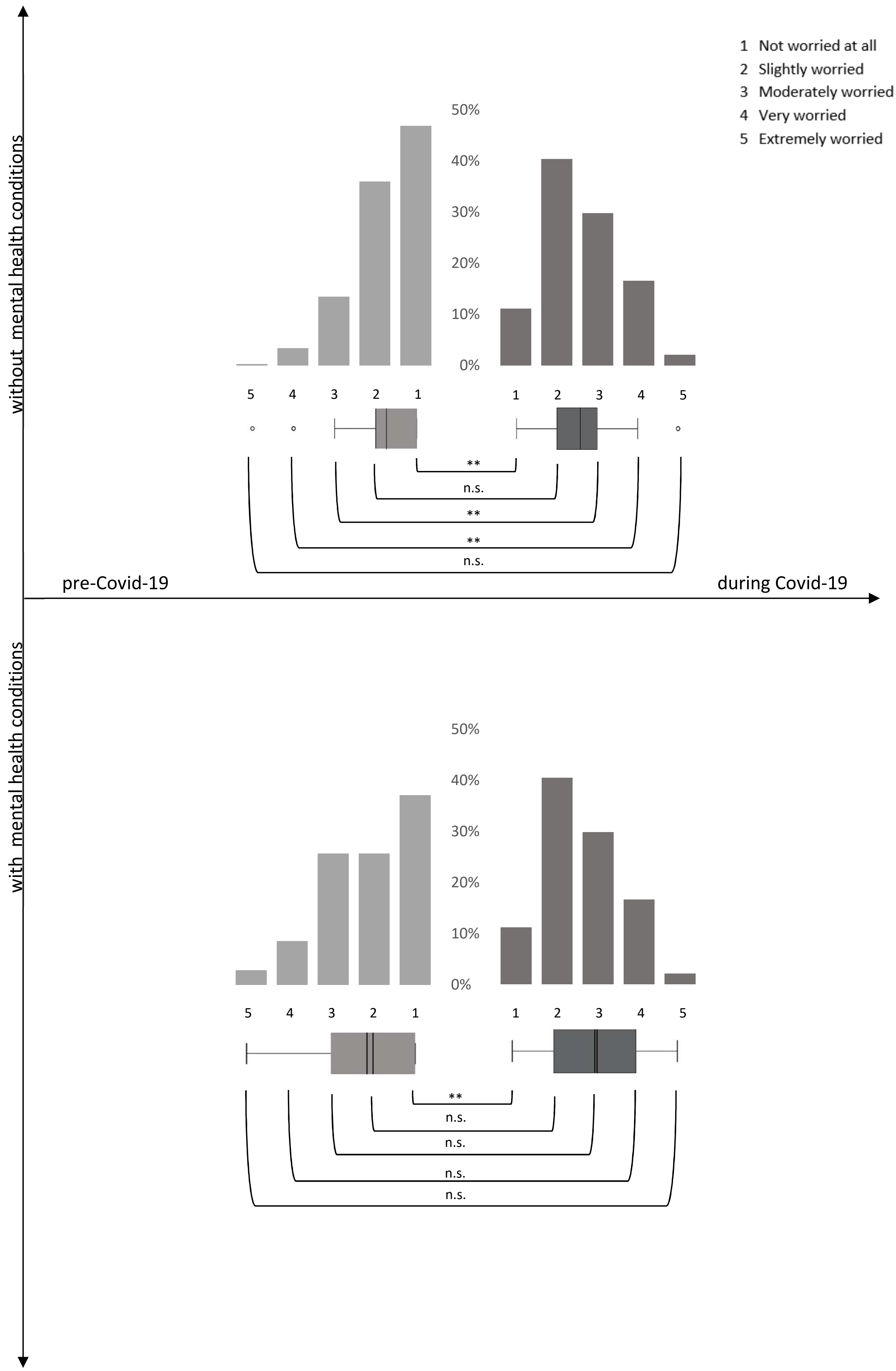

b) How happy versus sad were you?

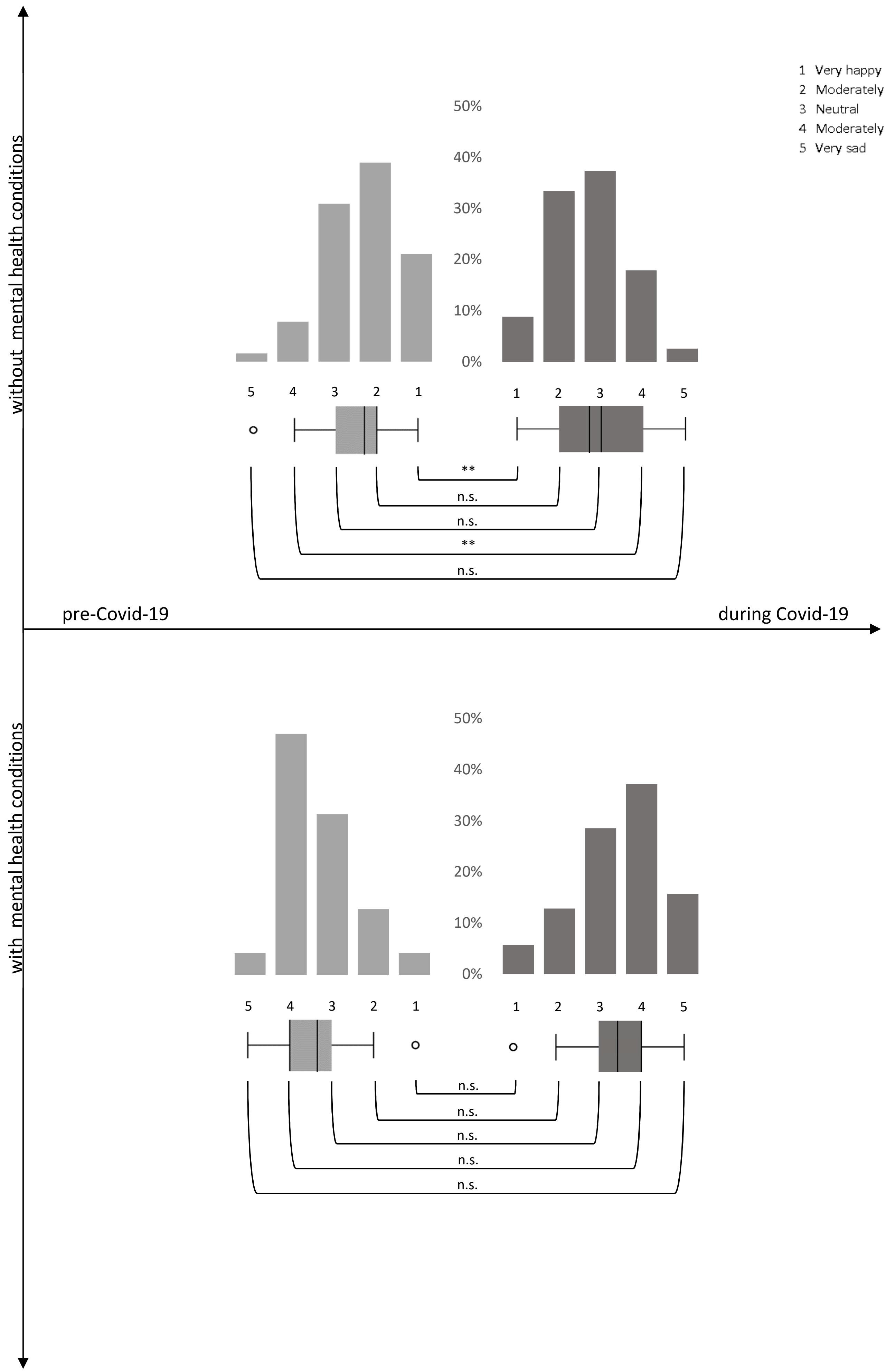

c) How much did you enjoy your usual activities?

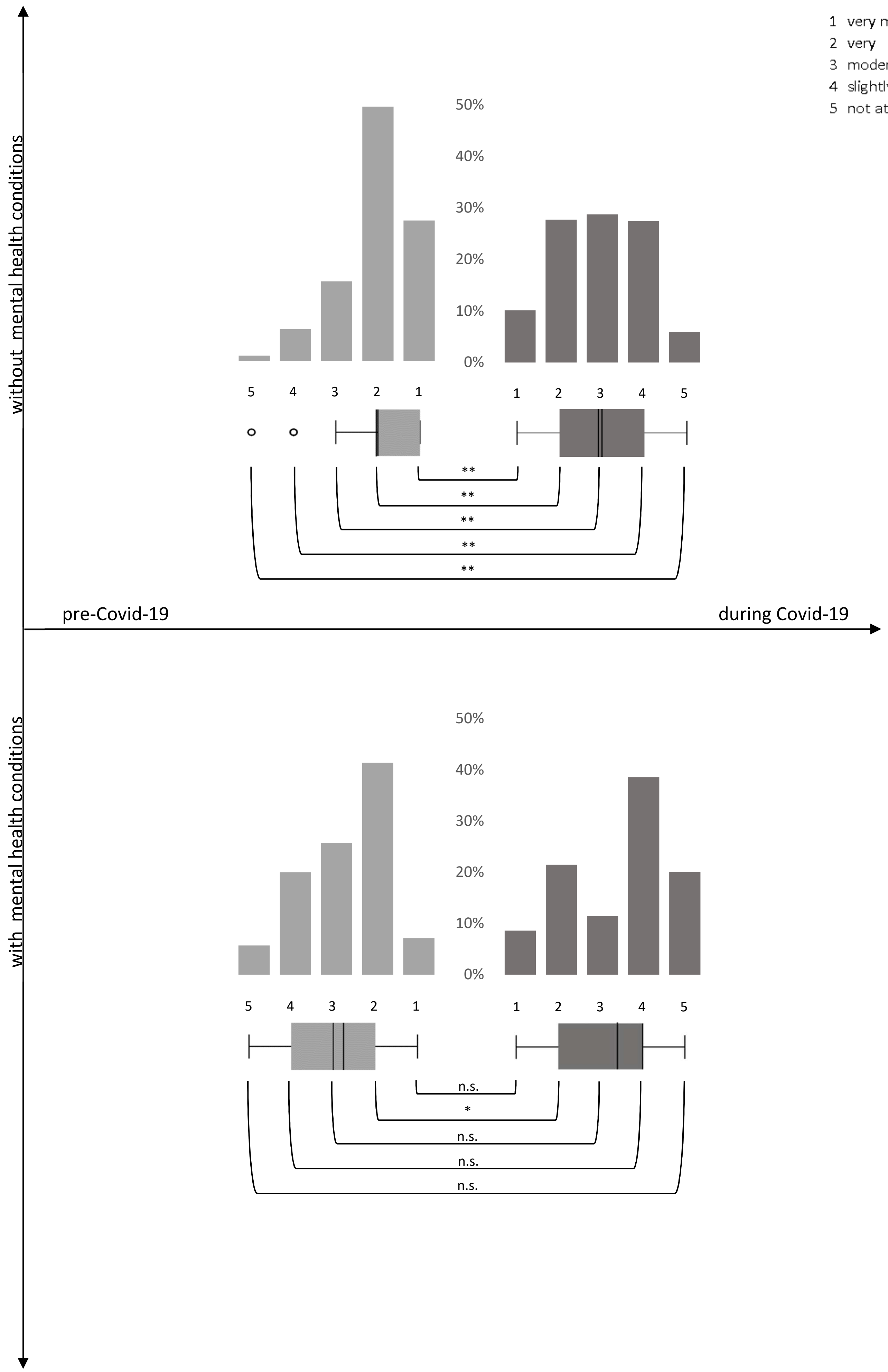

d) How relaxed versus anxious were you?

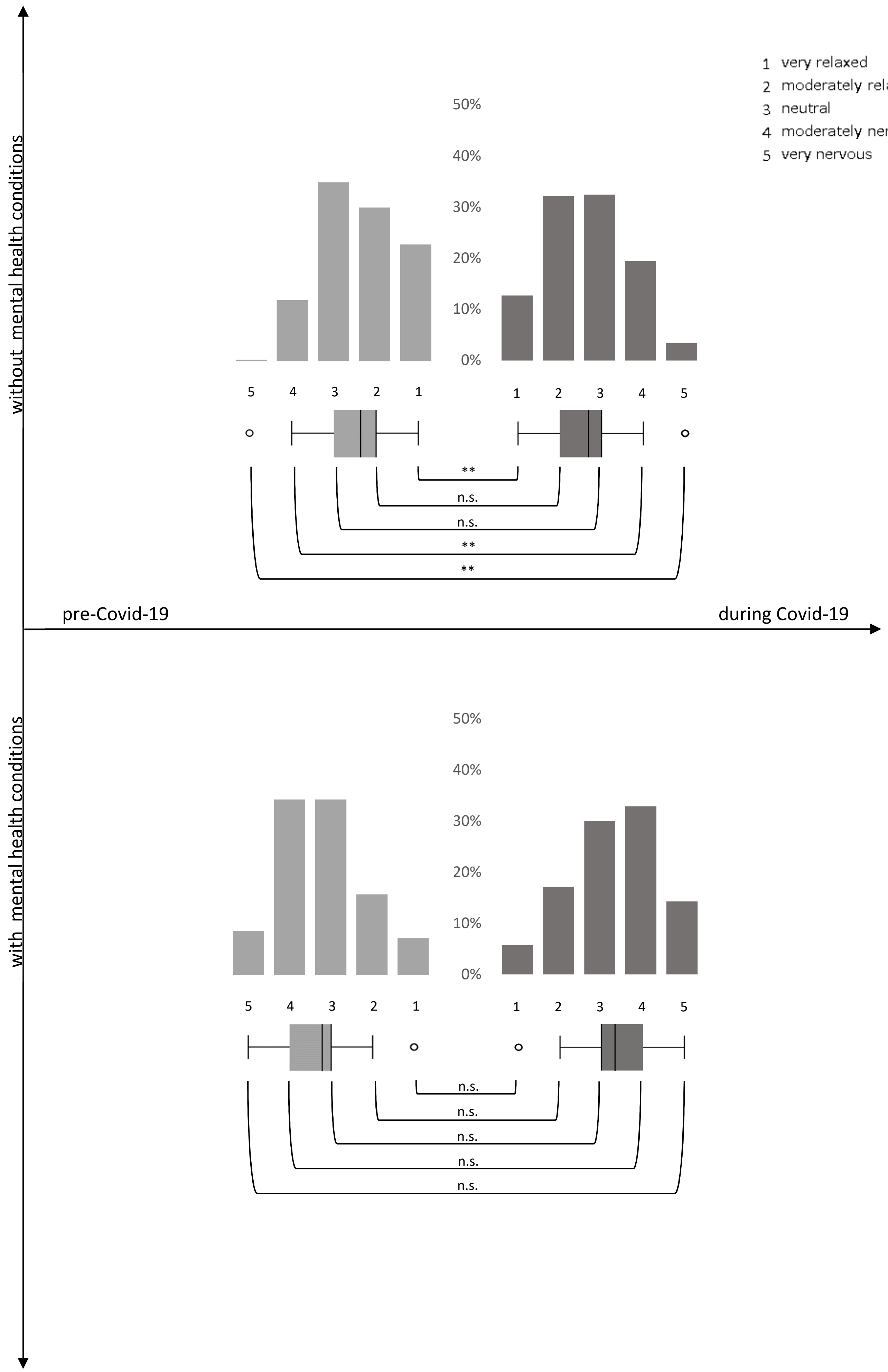

e) How fidgety or restless were you?

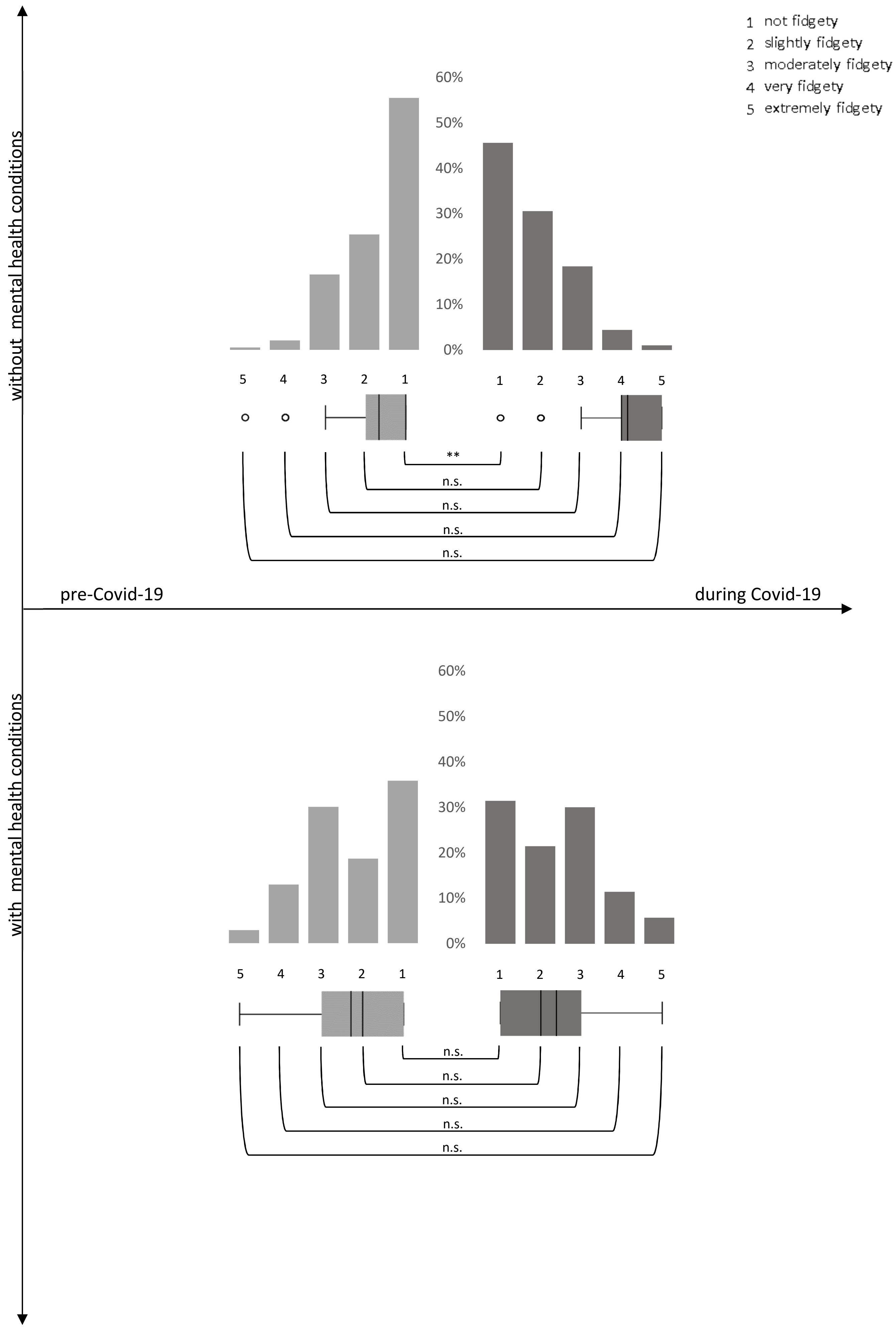

f) How fatigued or tired were you?

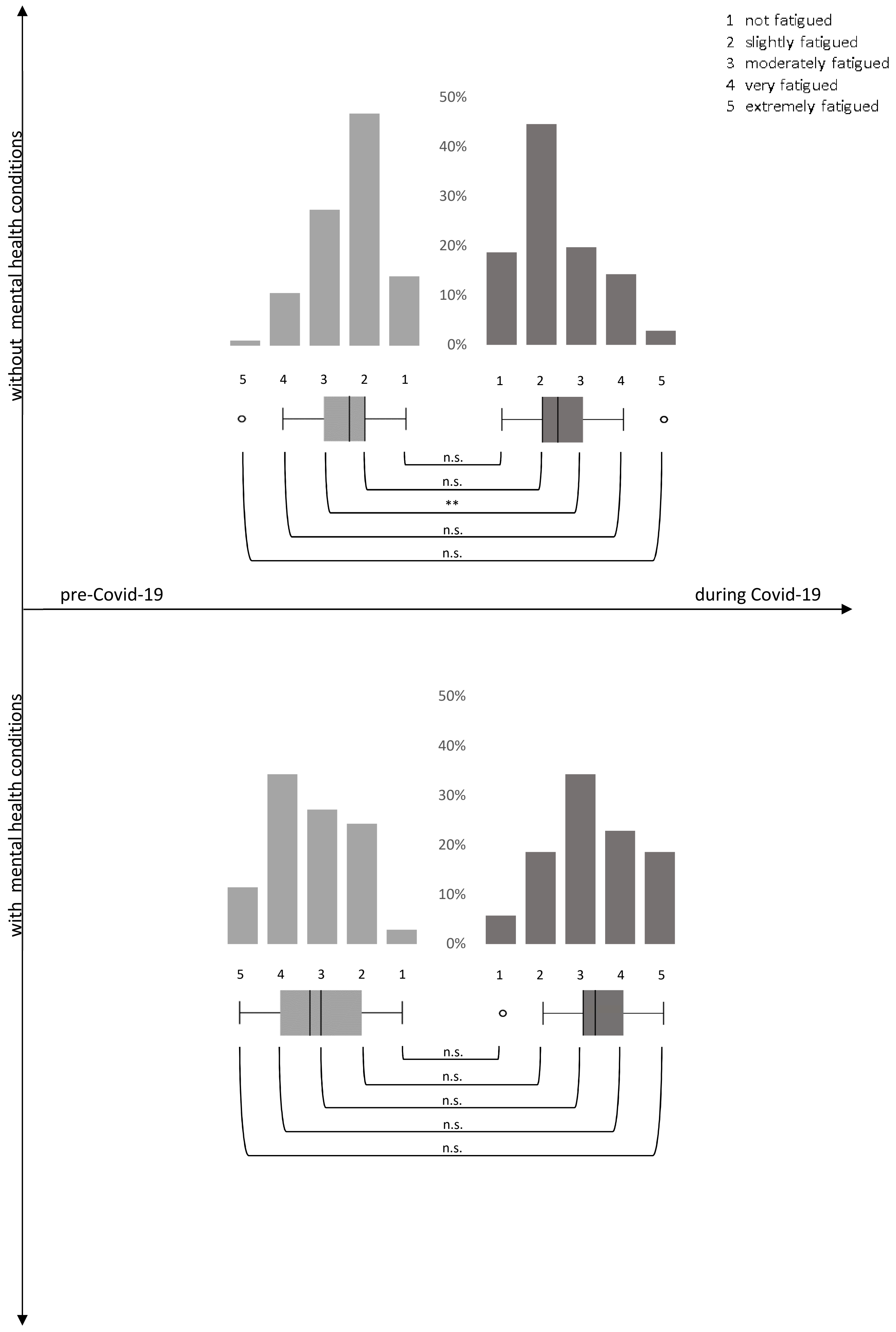

g) How well were you able to concentrate or focus?

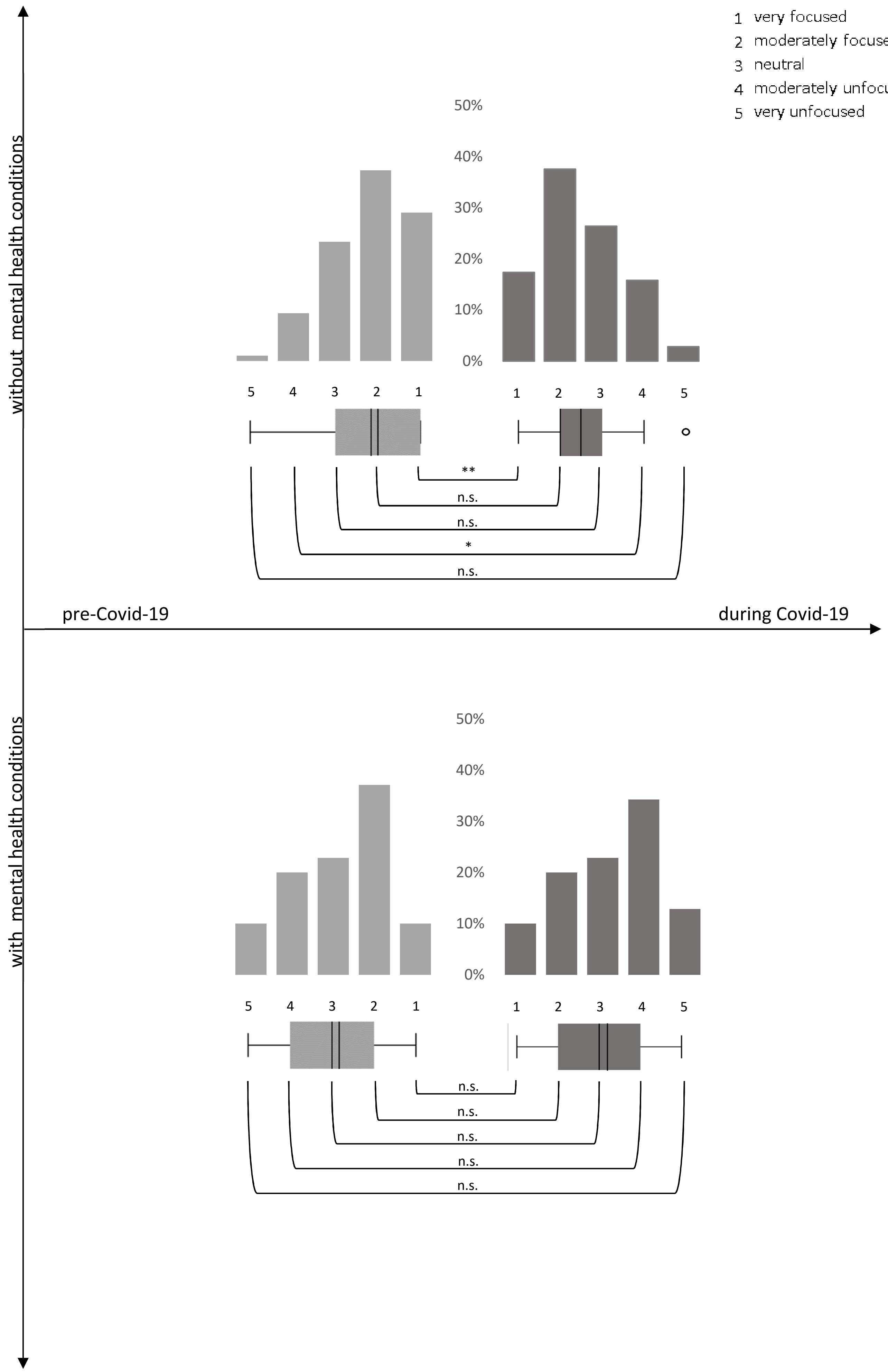

h) How irritable or easily angered were you?

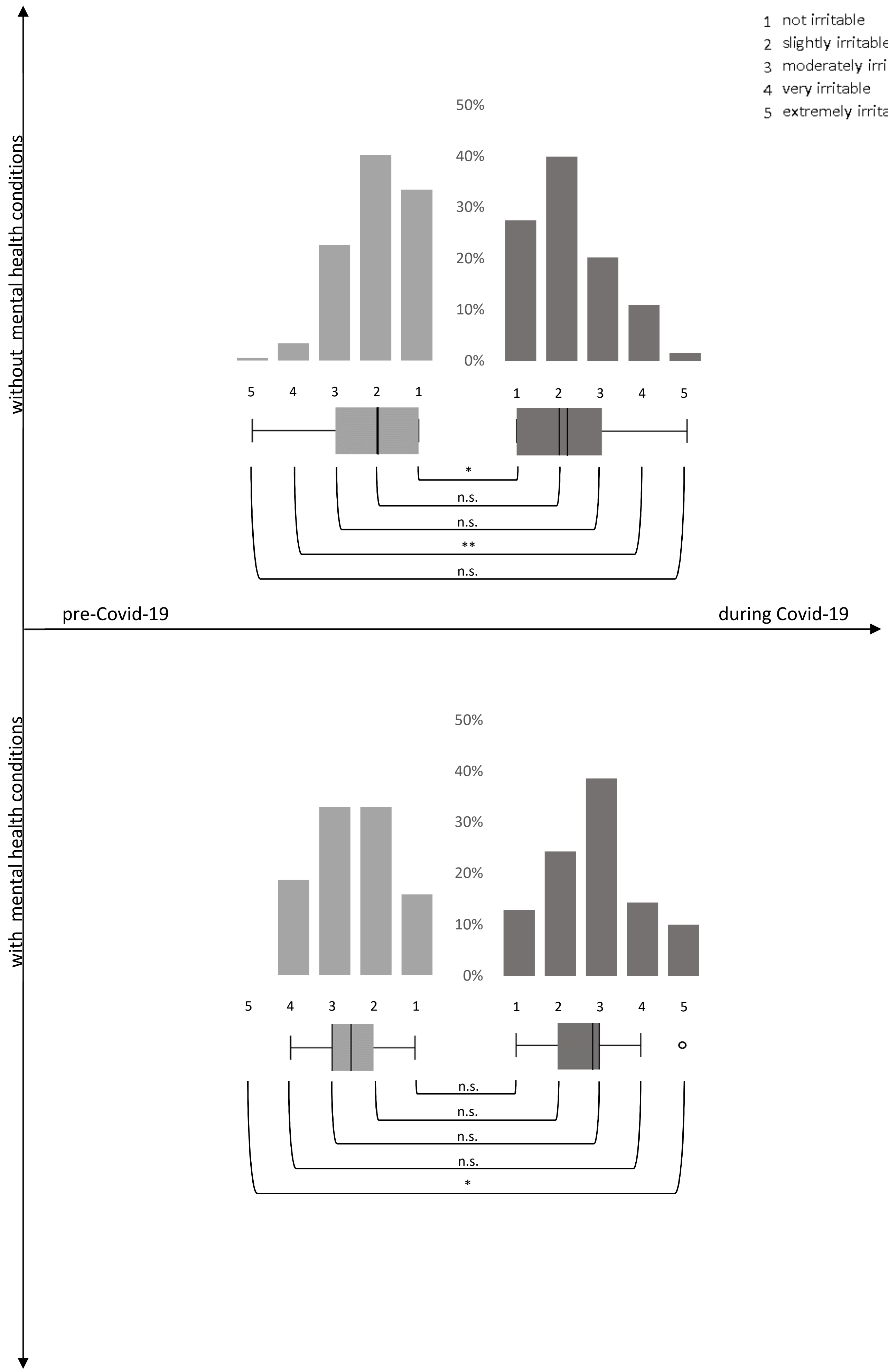

i) How lonely were you?

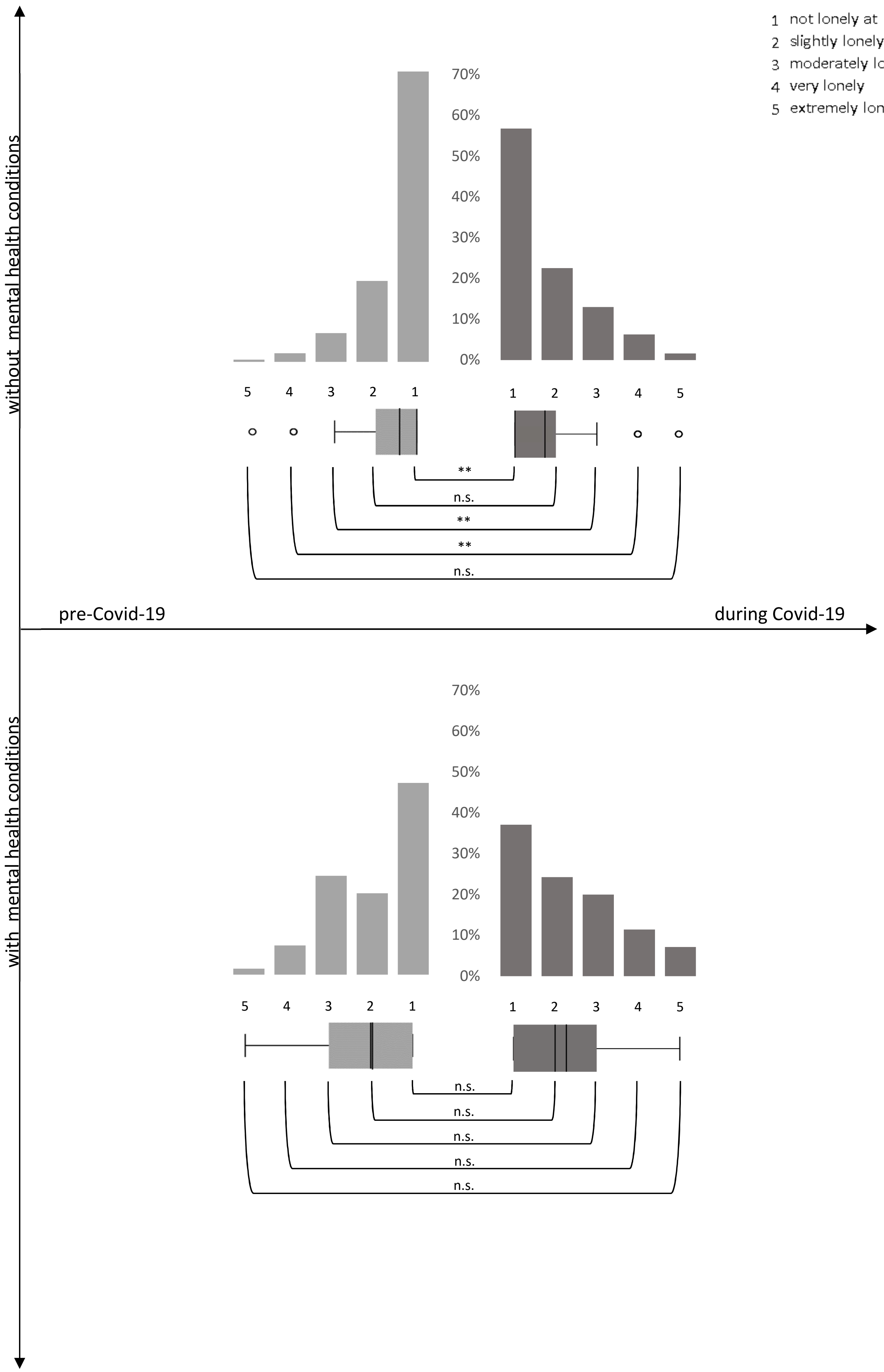

j) To what extent have you had negative thoughts?

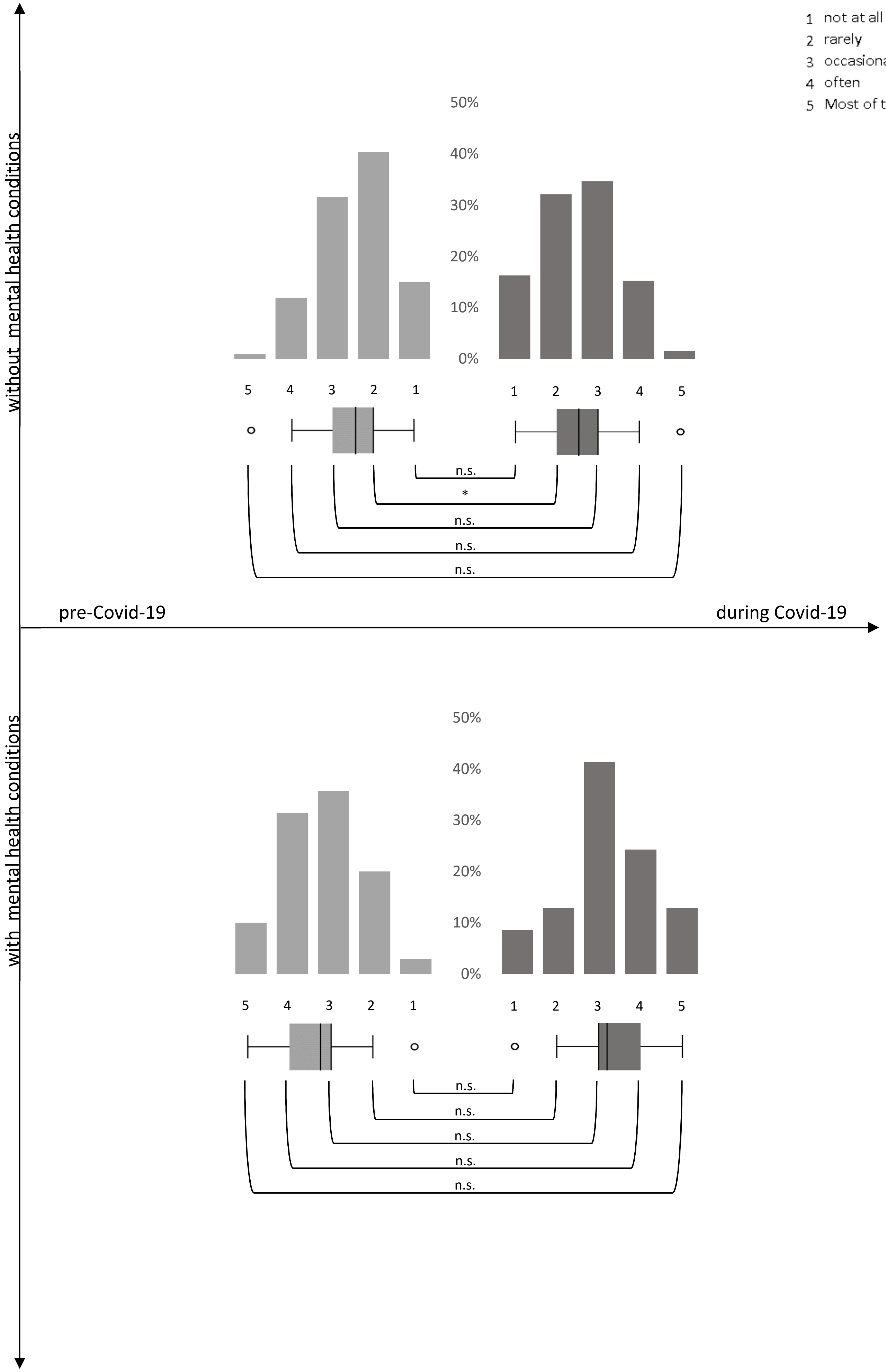

Supplement: Supplementary file 1 — Additional file 1. Frequencies of emotions and worries in adults. Frequencies of responses to all questions on emotions and worries in adults with and without mental health conditions (self-report). [file 13034_2021_363_MOESM1_ESM.pdf]
