# Supplementary material for: Changes in emotions and worries during the Covid-19 pandemic: an online-survey with children and adults with and without mental health conditions
Source: Child Adolesc Psychiatry Ment Health. 2021 Feb 21;15:11. doi: 10.1186/s13034-021-00363-9 (PMC7897360; doi:10.1186/s13034-021-00363-9)

a) How worried was your child generally?

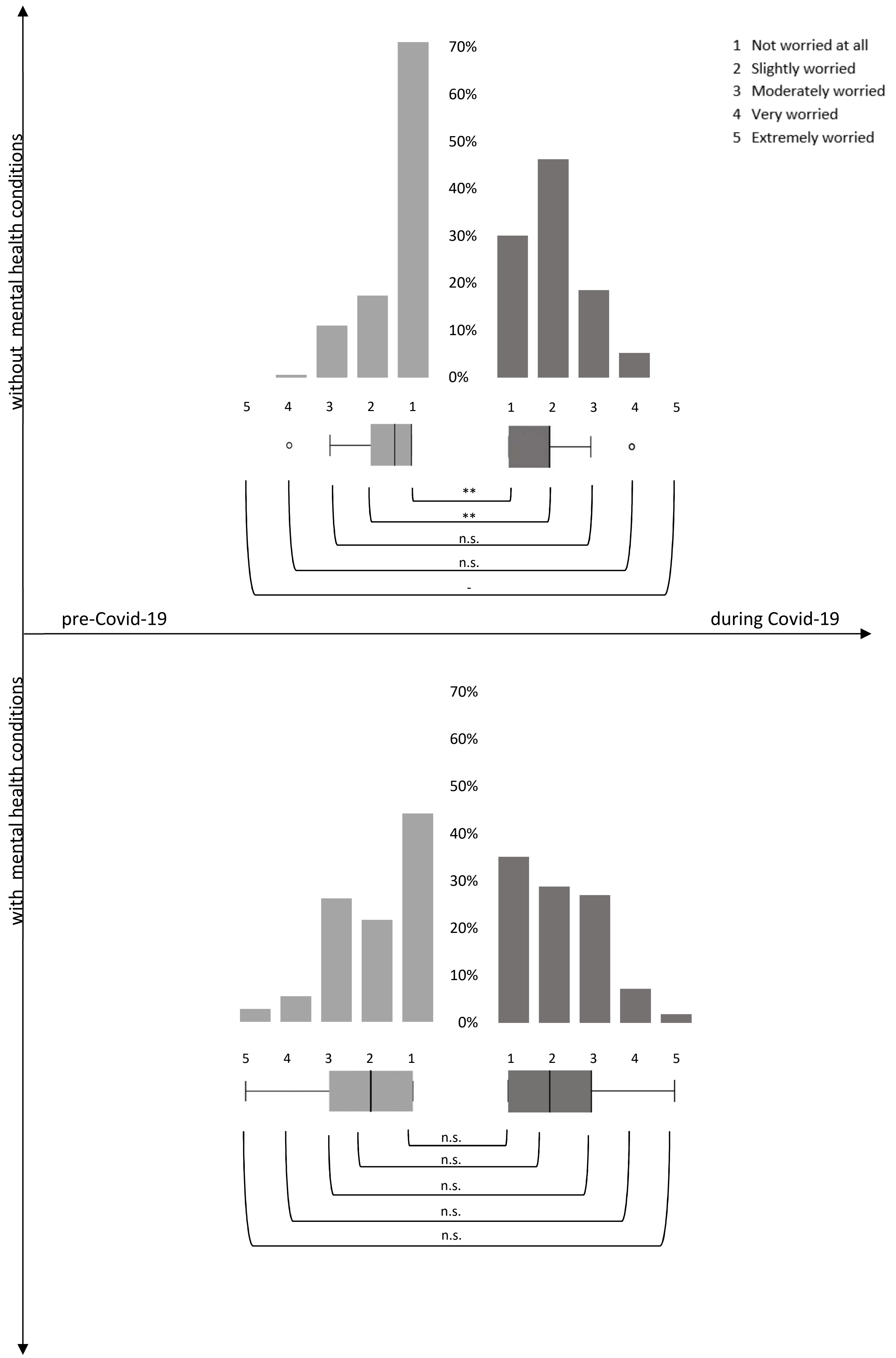

b) How happy versus sad was your child?

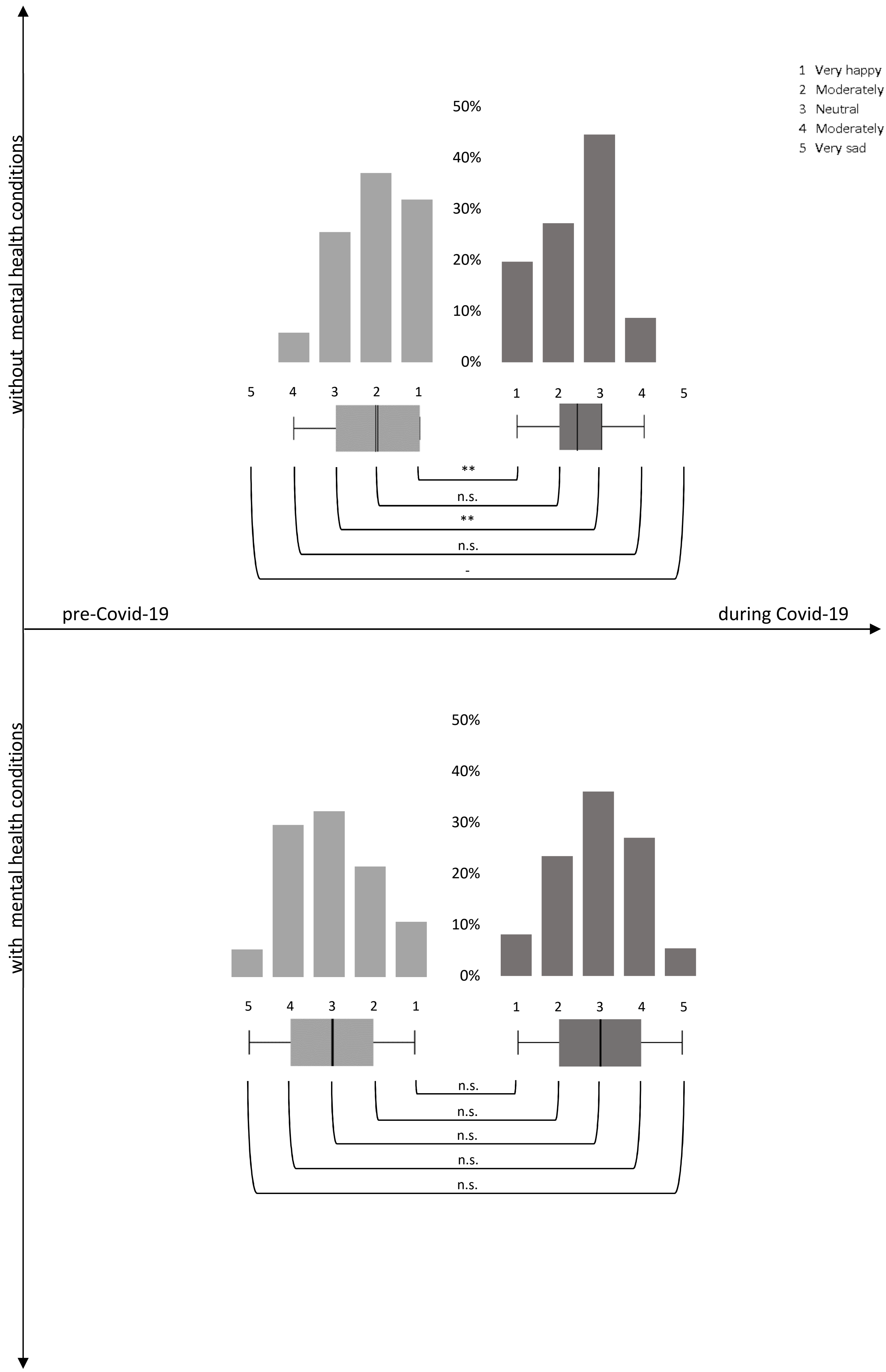

c) How much did your child enjoy their usual activities?

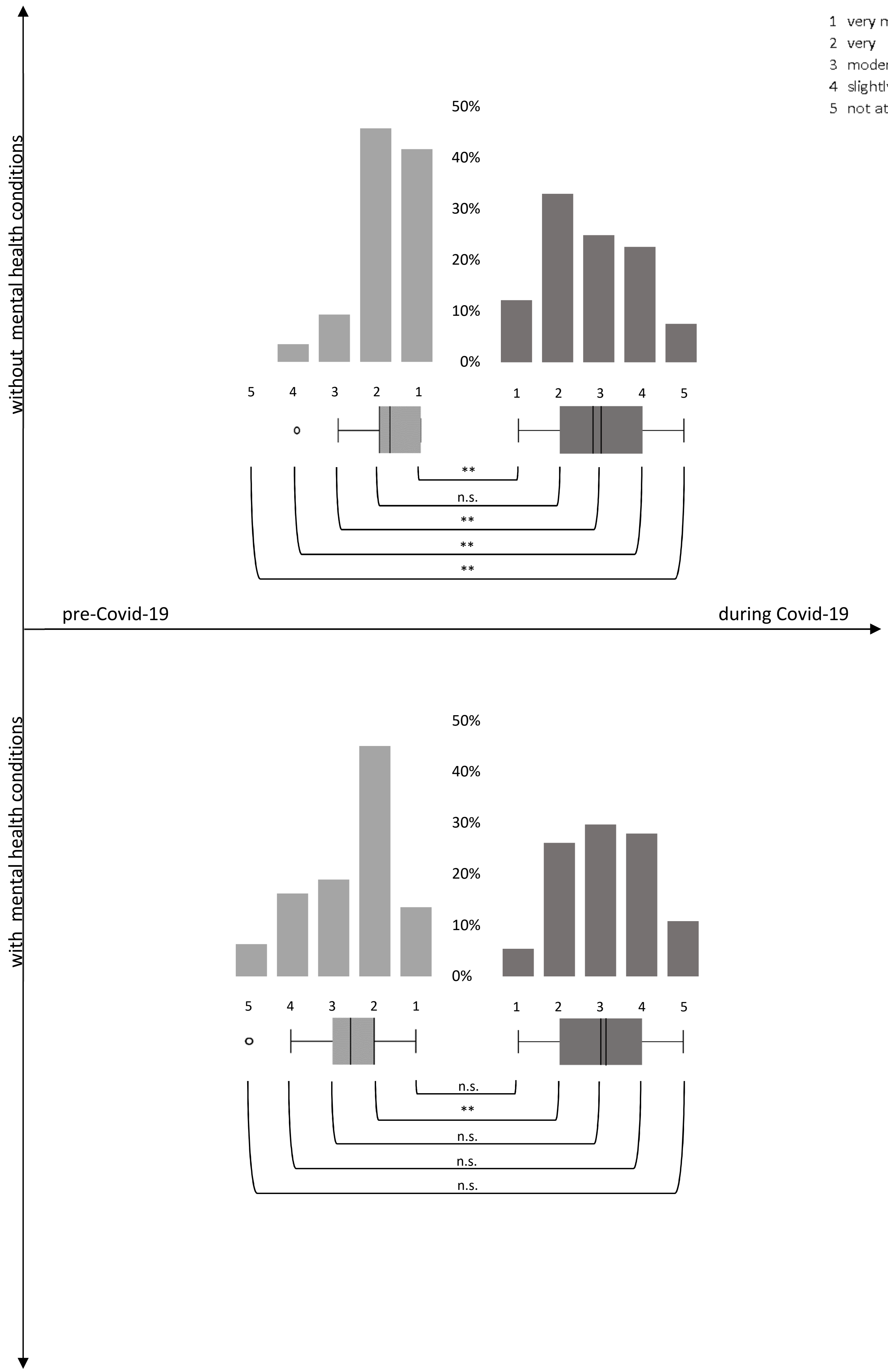

d) How relaxed versus anxious was your child?

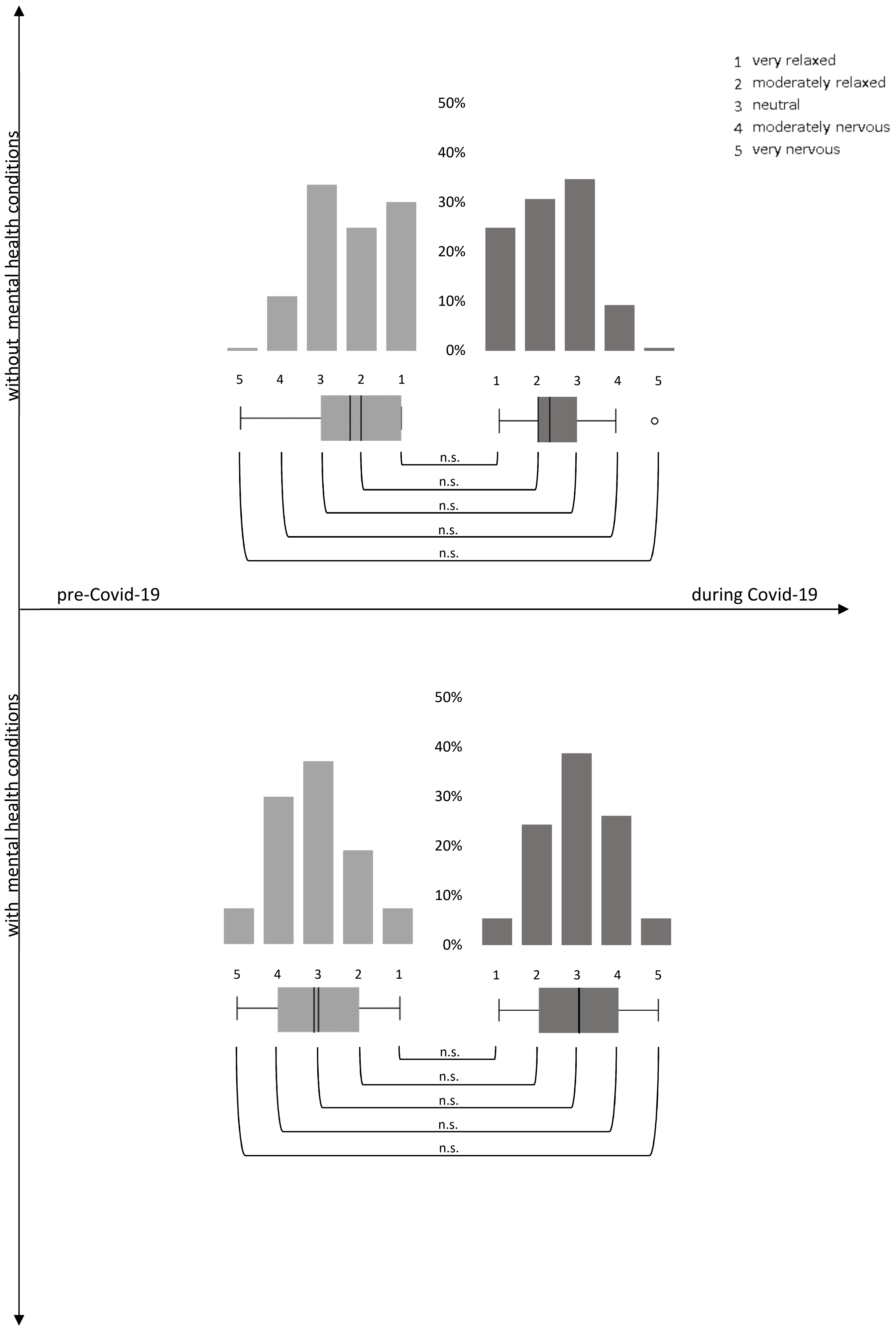

e) How fidgety or restless was your child?

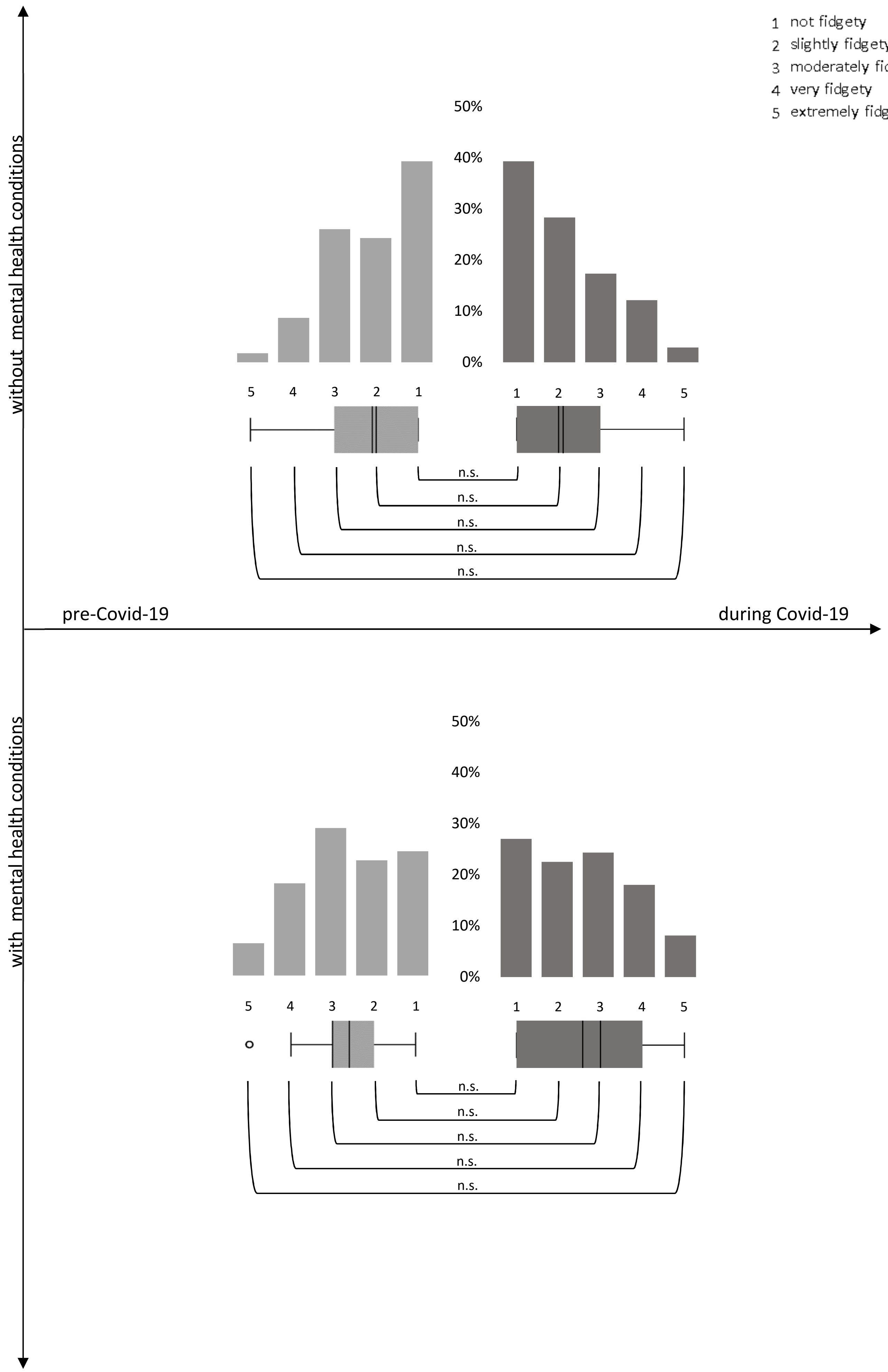

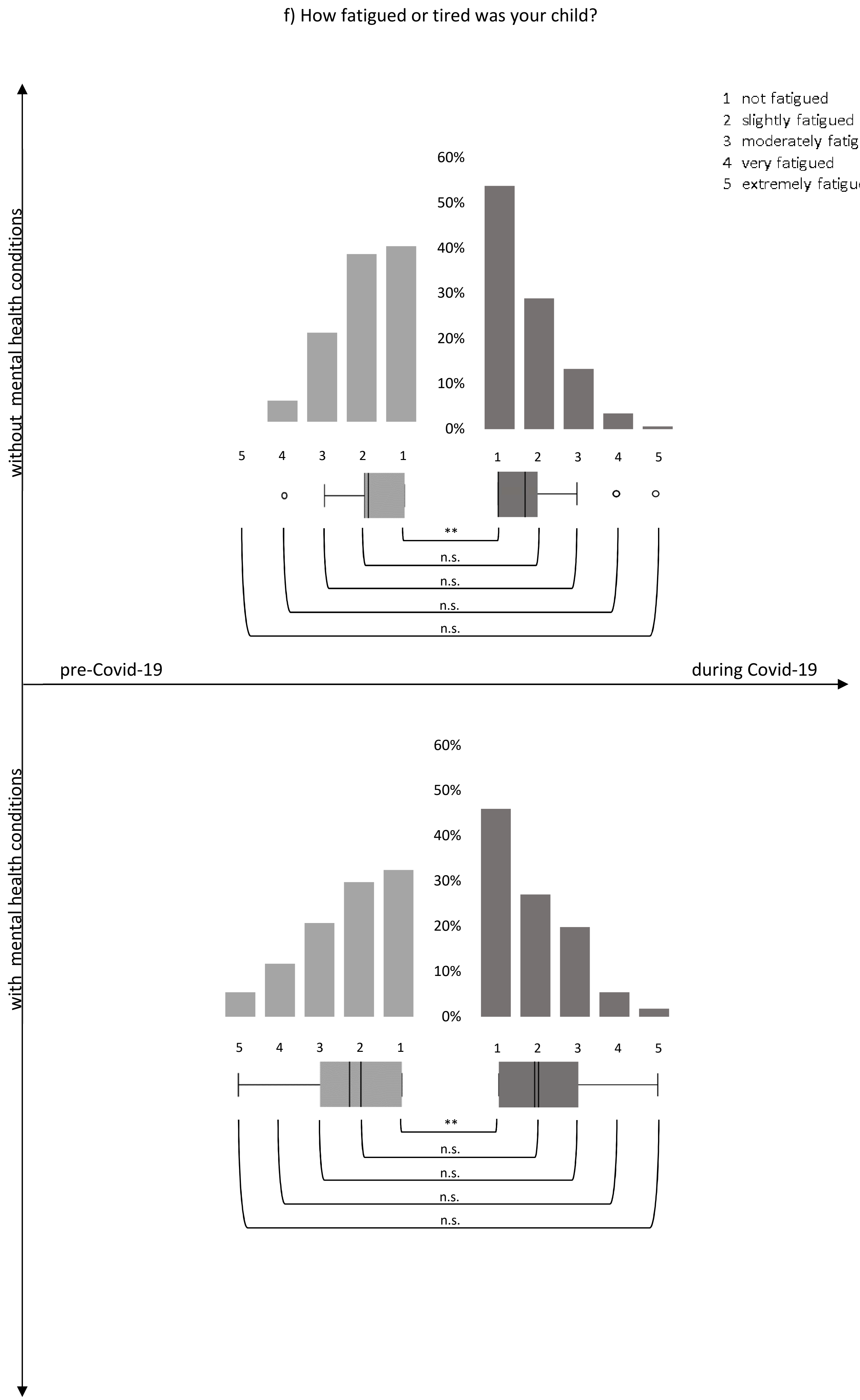

f) How fatigued or tired was your child?
1 not fatigued
2 slightly fatigued
3 moderately fatigued
4 very fatigued
5 extremely fatigued
without mental health conditions
with mental health conditions
pre-Covid-19
during Covid-19
60%
50%
40%
30%
20%
10%
0%
5 4 3 2 1
1 2 3 4 5
**
n.s.
n.s.
n.s.
n.s.

g) How well was your child able to concentrate or focus?

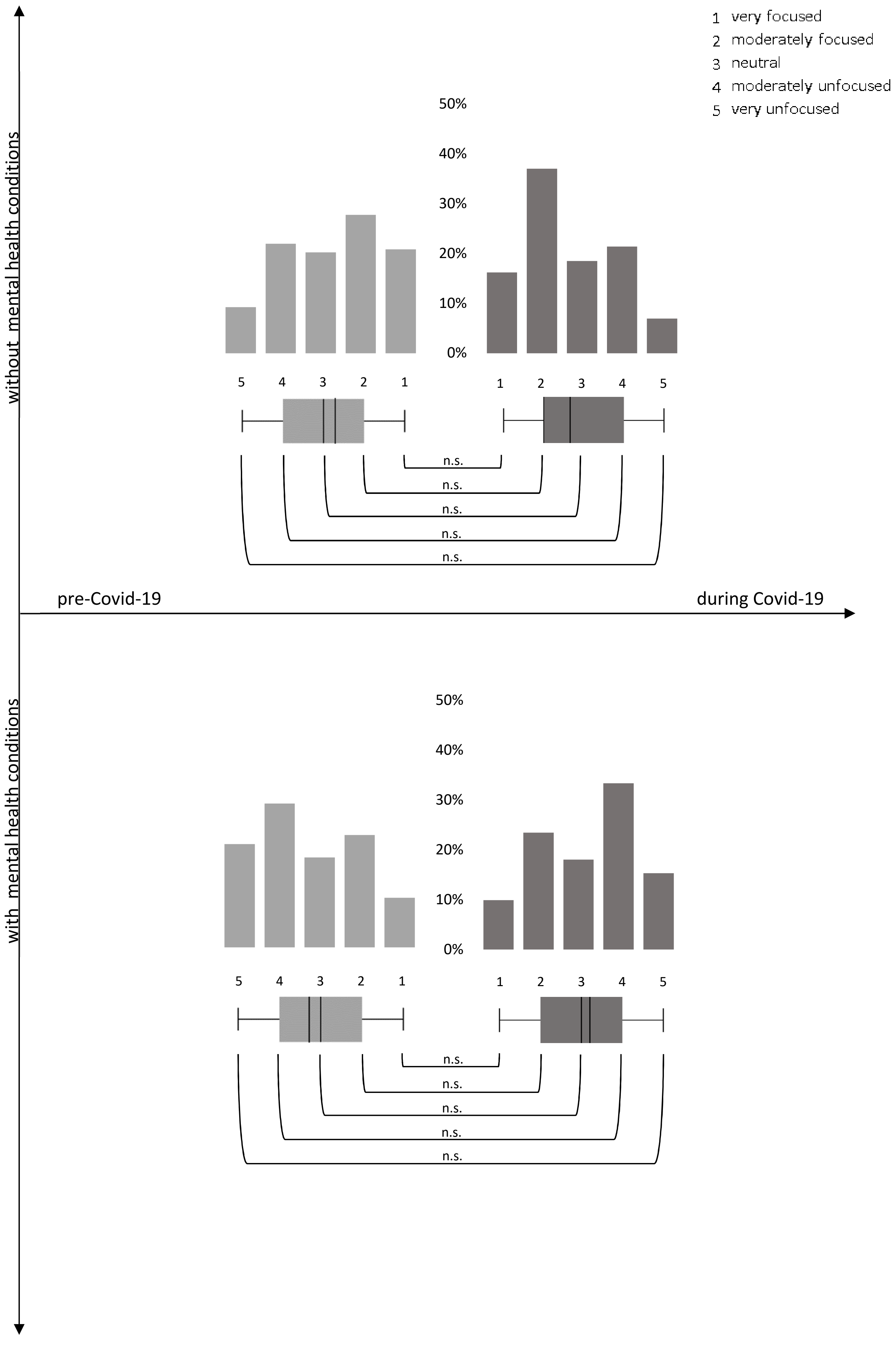

h) How irritable or easily angered was your child?

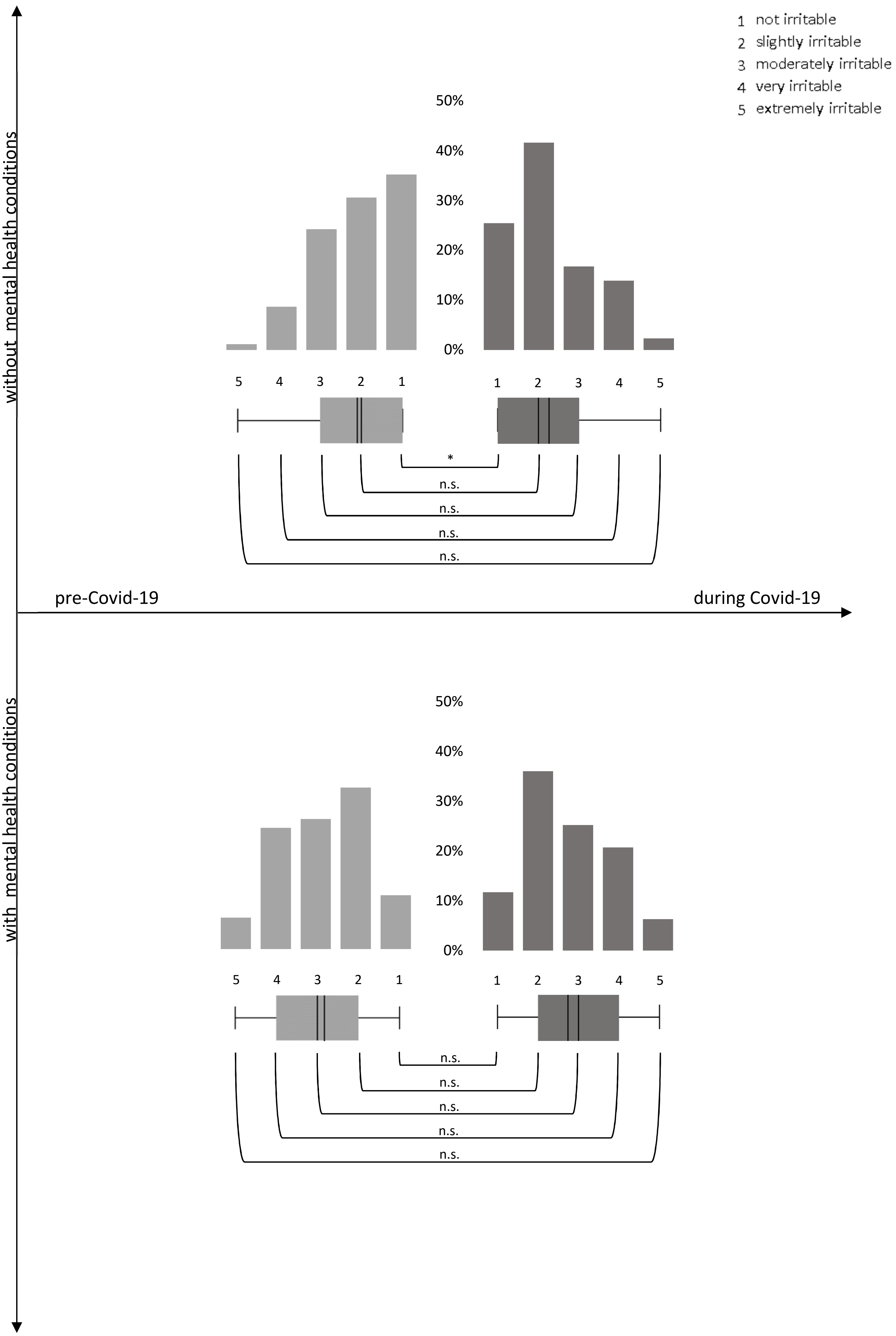

i) How lonely was your child?

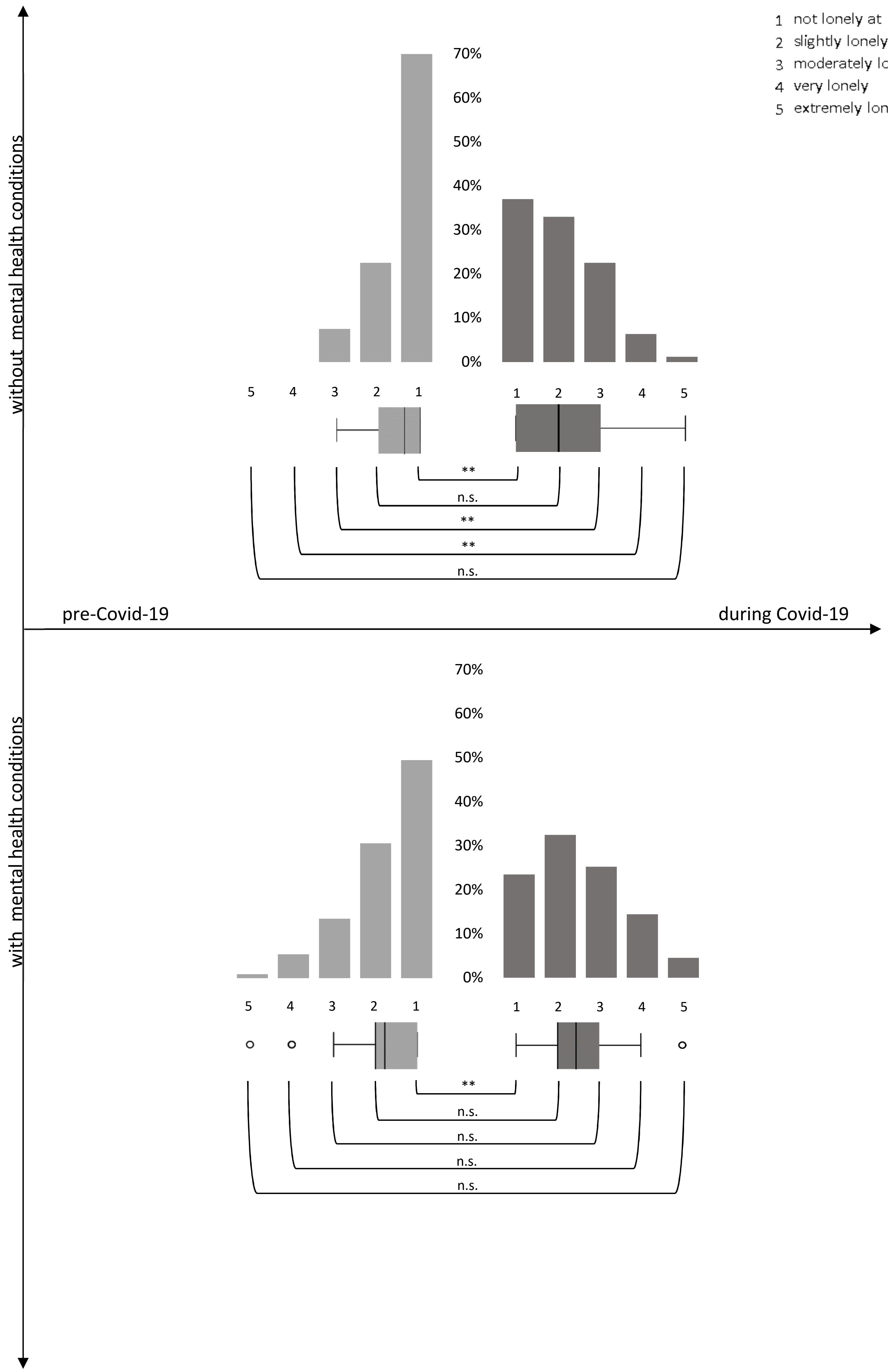

j) To what extent has your child had negative thoughts?

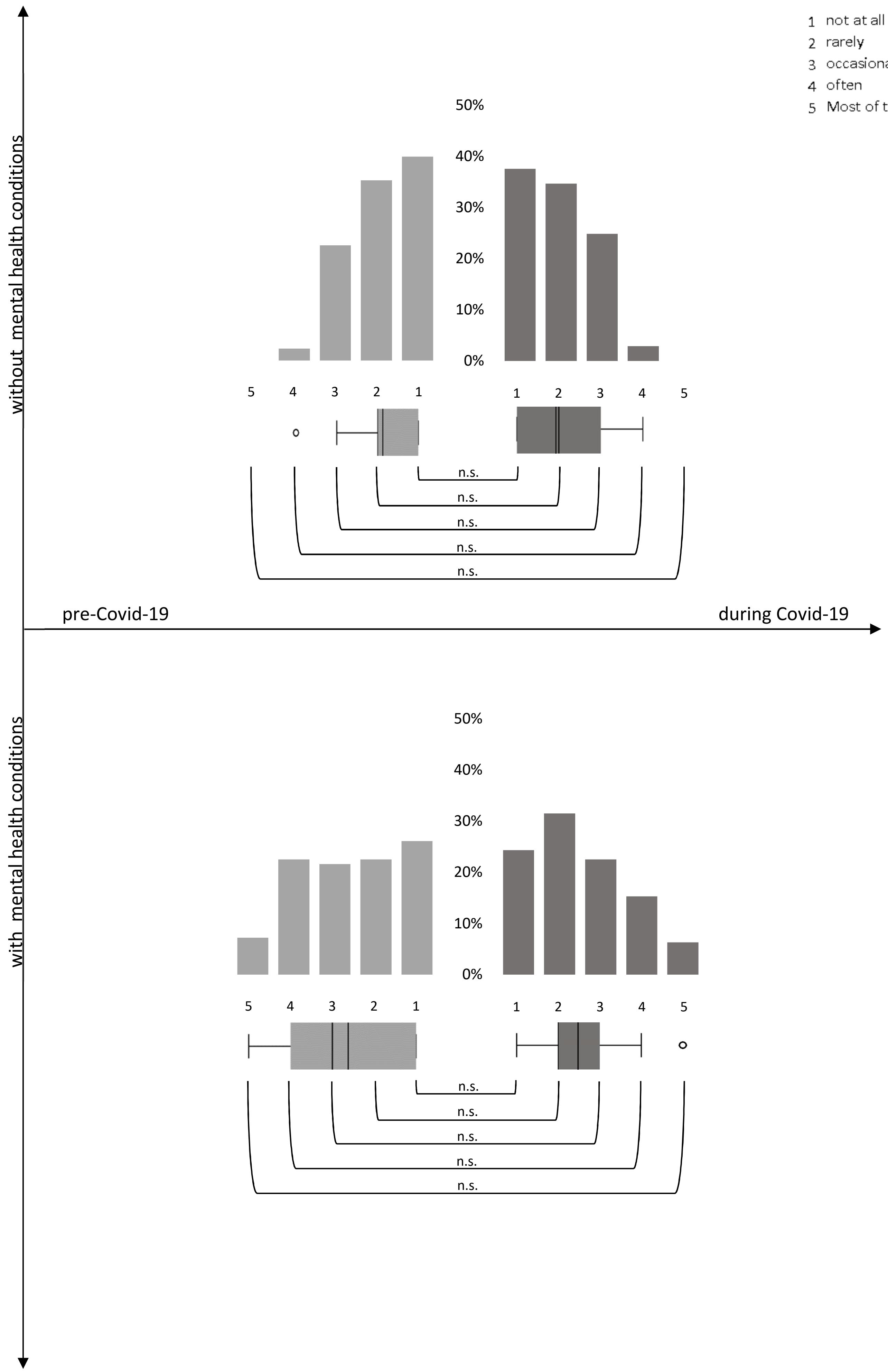

Supplement: Supplementary file 2 — Additional file 2. Frequencies of emotions and worries in children. Description of data: Frequencies of responses to all questions on emotions and worries in children with and without mental health conditions (parent-on-child-report). [file 13034_2021_363_MOESM2_ESM.pdf]
